# OA07 Upadacitinib exposure during pregnancy in seronegative Rheumatoid arthritis

**DOI:** 10.1093/rap/rkae117.007

**Published:** 2024-11-05

**Authors:** Chethana Ramakrishna, Christopher Holroyd

**Affiliations:** University Hospital Southampton, Southampton, United Kingdom; University Hospital Southampton, Southampton, United Kingdom

## Abstract

**Introduction:**

Targeted therapies have become increasingly prevalent in the management of rheumatoid arthritis (RA), with Janus kinase inhibitors (JAK inhibitors) gaining traction in clinical practice. Upadacitinib, a selective JAK1 inhibitor, is widely used for its efficacy in controlling RA. However, preclinical studies have demonstrated teratogenic effects associated with JAK inhibitors, raising concerns about their safety during pregnancy. Given the limited data on human exposure, current guidelines recommend the immediate discontinuation of JAK inhibitors upon pregnancy detection. This case report details an instance of Upadacitinib exposure during pregnancy.

**Case description:**

A 36-year-old woman was diagnosed with seronegative RA in January 2020. Her medical history is otherwise unremarkable. She has two children, aged four and two, both of whom were born after uneventful pregnancies. She has no history of spontaneous abortions.

At the time of her RA diagnosis, the patient was considering pregnancy and was initially started on sulfasalazine. However, due to the development of a rash, her treatment was switched to certolizumab injections. Despite this treatment, she exhibited only a partial response with ongoing disease activity, leading to a change to etanercept in August 2021.

When etanercept proved ineffective, adalimumab was considered in January 2022. Unfortunately, her response to adalimumab was limited. Consequently, she was started on Upadacitinib in July 2022. The patient responded well to Upadacitinib, achieving remission with normal inflammatory markers.

In April 2024, the patient reported being six weeks pregnant while on Upadacitinib. Upon confirmation of her pregnancy, Upadacitinib was discontinued at approximately four weeks of gestational age. The parents were informed about the potential risks of teratogenicity. After a detailed discussion with the high-risk pregnancy team, who provided reassurance, the patient decided to continue the pregnancy. She was subsequently started on certolizumab. Unfortunately, the patient experienced a miscarriage at 11 weeks of gestation.

**Discussion:**

Currently, there are no published human studies available in the literature regarding the effects of upadacitinib during pregnancy. It is crucial to encourage adequate contraception for women of childbearing potential and to verify a negative pregnancy status before initiating therapy. Women with reproductive potential should be advised to use effective contraception during upadacitinib therapy and for at least four weeks after discontinuation. If a patient becomes pregnant while on the drug, they should be informed of the potential risks to the foetus.

According to the 2019 product information on upadacitinib, animal studies have shown that the drug, when administered at exposures equal to or greater than approximately 1.6 to 15 times the maximum recommended human dose, resulted in dose-related increases in skeletal and cardiovascular malformations, increased post-implantation loss, and decreased foetal body weights. There are no controlled data on upadacitinib use during human pregnancy, and it is unknown whether the drug can cause foetal harm or adversely affect reproductive capacity in humans.

Data about pregnancy while on upadacitinib is limited. There are some unpublished data from the clinical trials and it was noted most of the patients had live births without congenital anomaly. Spontaneous abortions, elective terminations and ectopic pregnancies were among the other outcomes noted.

**Key learning points:**

• Due to issues such as primary or secondary treatment failures, hypersensitivity reactions, and adverse events, patients often require sequential trials of different agents in management of Rheumatoid Arthritis (RA). Janus kinase inhibitors (JAK inhibitors) have shown remarkable efficacy in managing RA over the last decade. Despite their widespread use, there is limited literature on the safety of upadacitinib exposure during pregnancy in humans. Most of the available data come from unexpected pregnancies documented during clinical trials.

• Animal studies indicate that upadacitinib can be harmful to a developing foetus. Furthermore, data suggest that women with RA who experience increased disease activity are at a heightened risk for adverse pregnancy outcomes, including preterm delivery, low birth weight, and infants who are small for gestational age.

• It is crucial to emphasize pregnancy contraindications during follow-up appointments for patients on these medications. Access to a medical obstetrician team is invaluable in managing such cases.

• While some case reports have documented positive pregnancy outcomes with other JAK inhibitors, such as tofacitinib and baricitinib, there remains a significant gap in knowledge regarding upadacitinib. This case report adds to the limited data on the potential risks associated with upadacitinib exposure during pregnancy. Comprehensive and targeted studies are necessary to better understand the safety of this class of drugs during pregnancy and lactation.

